# Uncommon properties of lipid biosynthesis of isolated plastids in the unicellular red alga *Cyanidioschyzon merolae*

**DOI:** 10.1002/2211-5463.12551

**Published:** 2018-12-04

**Authors:** Natsumi Mori, Takashi Moriyama, Naoki Sato

**Affiliations:** ^1^ Department of Life Sciences Graduate School of Arts and Sciences The University of Tokyo Japan

**Keywords:** *Cyanidioschyzon merolae*, isolated plastids, radiolabeling experiment, red alga, stable isotope, stearoyl‐ACP desaturase

## Abstract

Red algae are a large group of photosynthetic eukaryotes that diverged from green algae over one billion years ago, and have various traits distinct from those of both green algae and land plants. Although most red algae are marine species (both unicellular and macrophytic), the Cyanidiales class of red algae includes unicellular species which live in hot springs, such as *Cyanidioschyzon merolae*, which is a model species for biochemical and molecular biological studies. Lipid metabolism in red algae has previously been studied in intact cells. Here, we present the results of radiolabeling and stable isotope labeling experiments in intact plastids isolated from the unicellular red alga *C. merolae*. We focused on two uncommon features: First, the galactose moiety of monogalactosyldiacylglycerol was efficiently labeled with bicarbonate, indicating that an unknown pathway for providing UDP‐galactose exists within the plastid. Second, saturated fatty acids, namely, palmitic and stearic acids, were the sole products of fatty acid synthesis in the plastid, and they were efficiently exported. This finding suggests that the endoplasmic reticulum is the sole site of desaturation. We present a general principle of red algal lipid biosynthesis, namely, ‘indigenous C18 fatty acids are neither desaturated nor directly utilized within the plastid'. We believe that this is valid in both *C. merolae* lacking polyunsaturated fatty acids and marine red algae with a high content of arachidonic and eicosapentaenoic acids.

**Abbreviations**
ACP: acyl carrier protein
DAG: diacylglycerol
DGDG: digalactosyldiacylglycerol
ER: endoplasmic reticulum
FAME: fatty acid methyl ester
FFA: free fatty acid
G3P: glycerol 3‐phosphate
KAS: 3‐ketoacyl‐(acyl‐carrier‐protein) synthase
MGDG: monogalactosyldiacylglycerol
PA: phosphatidic acid
PC: phosphatidylcholine
PE: phosphatidylethanolamine
PG: phosphatidylglycerol
PI: phosphatidylinositol
RP‐TLC: reversed‐phase argentation thin‐layer chromatography
SQDG: sulfoquinovosyldiacylglycerol

Red algae are a large group of photosynthetic eukaryotes originated from the primary endosymbiosis (Archaeplastida) that diverged from green algae and glaucophytes more than a billion years ago. They have various traits distinct from green algae and land plants, such as the presence of phycobiliproteins as light‐harvesting complex in photosynthesis. *Pyropia yezoensis*, known as ‘Nori' in Japanese traditional food is rich in arachidonic (20:4) and eicosapentaenoic acids (20:5) as membrane lipid components 1 (each fatty acid is designated by a combination of number of carbons [X] and number of double bonds [Y], such as X:Y.). Although most red algae are marine species (both unicellular and macrophytic), Cyanidiales includes unicellular species living in hot springs. *Cyanidioschyzon merolae*, a representative, species of Cyanidiales, has been studied as a model red alga 2, and we have characterized metabolism of its component lipids in experiments using intact cells 1, 3, 4, 5, 6, 7. In spite of the lack of polyunsaturated fatty acids, we consider this alga useful because it contains a single plastid within the cell. We then presented a hypothesis that the apparent differences in fatty acid composition in this alga and macrophytes could be understood in a common framework focusing on the role of plastid 1.

Plastids are one of the major compartments of lipid metabolism in plants and algae, in which fatty acids and plastid membrane lipids are synthesized, such as monogalactosyldiacylglycerol (MGDG), digalactosyldiacylglycerol (DGDG), sulfoquinovosyldiacylglycerol (SQDG), and phosphatidylglycerol (PG) 8, 9, 10. In plants, plastid is the sole site of long‐chain fatty acid synthesis 11, and this is believed to be true for most algae 5, 12, 13. The site of desaturation is, however, variable depending on fatty acids and double‐bond position to be inserted. The desaturation for the synthesis of polyunsaturated fatty acids found in MGDG takes place in both plastids and endoplasmic reticulum (ER) in plants. Namely, there are two pathways for the synthesis of diacylglycerol (DAG) moiety of MGDG, one completed within the plastids, and the other involving cooperation of plastids and ER. The two pathways have been traditionally called ‘prokaryotic' and ‘eukaryotic' pathways 14, 15. This naming has a connotation that plastids originate from ancestral cyanobacteria. As we recently pointed out 16, most enzymes involved in the ‘prokaryotic' pathway did not originate from cyanobacteria, and most probably originated from the gene pool of ancestral eukaryotes. That is why we try to avoid using the words, prokaryotic and eukaryotic, as pathway names in the present study. In any way, there are two distinct pathways in plants and probably green algae. The situation in red algae, however, might be different.

We previously found in *C. merolae* ‘coupled pathway' for the synthesis of linoleoyl/palmitoyl species of MGDG 3, 5, in which palmitic acid (16:0) is supplied from the inside of plastids whereas linoleic acid (18:2) is provided from outside (probably from ER) after desaturation. This molecular species was not synthesized by sequential desaturation from stearoyl/palmitoyl species through oleoyl/palmitoyl species (in the following, stearic and oleic acids are abbreviated as 18:0 and 18:1, respectively). Both 18:2 and 16:0 are simultaneously required to produce a single molecule of MGDG, probably in the envelope membranes of plastids. This situation is different from that in plants and green algae in which there are two different pools of DAG, one produced within the plastid and the other imported from ER. In the marine red alga *Porphyridium purpureum*, MGDG is also likely to be synthesized by the coupled pathway from 16:0 and 20:4, which are generated in the plastids and the extraplastidial compartment, respectively 17. This similarity is an important point favoring the use of *C. merolae* as a model of all red algae, including marine species.

A fundamental reason for this peculiar pathway was obtained by comparative genomic analysis: In red algae, namely, there is no stearoyl‐acyl carrier protein (ACP) desaturase that is ubiquitous in land plants and green algae 1, 3, in which 18:0 synthesized by fatty acid synthase (FAS) is rapidly desaturated to 18:1 within the plastids 12, 18. We have to verify the fate of 18:0 synthesized within the plastids using isolated red algal plastids, whether 18:0 rather than 18:1 is a major product.

In plants, lipid metabolism in chloroplasts was studied by radiolabeling experiments using isolated plastids in spinach 19, 20, 21, 22, pea 23, lettuce 24, and olive 25. On the other hand, in most red algae (many of them are marine macrophytes), methods of isolation of intact plastids are not available. Tracer experiments using radioisotopes have been performed in intact cells for the analysis of lipid metabolism in red algae 17, 26, 27, 28. In isolated spinach chloroplasts, [2‐^14^C]acetate and [^14^C]bicarbonate were tested for incorporation into lipids via fatty acid synthesis and photosynthesis, respectively. Incorporation of [2‐^14^C]acetate into fatty acids was about 10 times higher than that of [^14^C]bicarbonate 20, 29, 30, which is explained by the fact that cytosolic enzymes, such as phosphoglycerate mutase and enolase, are required to synthesize acetyl‐CoA from photosynthates in chloroplasts 31. Galactolipid synthesis activity was very low in isolated chloroplasts without additional supply of UDP‐galactose, which is synthesized in the cytosol in land plants 32.

We established the method of isolating metabolically active, highly purified plastids from high‐density culture of *C. merolae* cells 33. In the present study, we applied this technique to radiolabeling and stable isotope labeling experiments to obtain insights into the synthesis of fatty acids and lipids in red algal plastids. Intact plastids isolated from *C. merolae* cells were incubated with labeled acetate or bicarbonate, and then incorporation of ^14^C or ^13^C into fatty acids and lipids was analyzed. We found some interesting characteristics of labeling which are not common in experiments with plant chloroplasts. We will discuss these results in our framework of hypothesis on the general lipid metabolism in red algae.

## Materials and methods

### Culture condition of *C. merolae*

*Cyanidioschyzon merolae* 10D 34 was grown in 2 × Allen's medium 35 in a shaking flask under continuous light (30 μmol·m^−2^·s^−1^) at 40 °C.

### Isolation of plastids

*Cyanidioschyzon merolae* cells were grown to OD_750_ = 2 in 500 mL of 2 × Allen's medium at 40 °C with aeration by 1% CO_2_ under continuous light (50 μmol·m^−2^·s^−1^) and then collected by centrifugation (1600 ***g**,* 5 min). Precipitated cells were resuspended in 50 mL of fresh medium to OD_750_ = 10 and allowed to grow in a flat‐plate (2‐mm in thickness) culture apparatus 4 at 42 °C with aeration by 1% CO_2_ under light (250 μmol·m^−2^·s^−1^) for 12 h. All subsequent procedures were performed at 4 °C. *C. merolae* cells were harvested by centrifugation (1600 ***g***, 10 min) and suspended in Grinding buffer 1 (pH 7.5; 100 mm sorbitol, 30 mm HEPES‐KOH, 2 mm EDTA, 1 mm MgCl_2_, 1 mm MnCl_2_, 0.1% BSA). The cells were broken by French Pressure Cell (Ohtake, Tokyo, Japan) at 280 kg·cm^−2^ (4000 psi) and immediately mixed with an equal volume of Grinding buffer 2 (pH 7.5; 330 mm sorbitol, 30 mm HEPES‐KOH, 2 mm EDTA, 1 mm MgCl_2_, 1 mm MnCl_2_, 0.1% BSA) to recover isotonic osmotic pressure. After addition of RNase A (20 μg·mL^−1^) and DNase I (100 μg·mL^−1^) to digest RNA and released nuclear DNA, the lysate was incubated on ice for 60 min. It was then filtered through a nylon mesh (10 μm nominal pore size) and a Miracloth (Calbiochem, La Jolla, CA, USA). After centrifugation (2000 ***g**,* 10 min), the precipitate was suspended in 20% Percoll (GE Healthcare, Buckinghamshire, UK) in Grinding buffer 2 and then layered on top of a two‐step gradient consisting of 40% and 80% Percoll in Grinding buffer 2. After centrifugation (2000 ***g**,* 60 min), intact plastids were recovered at the 40%/80% interface. Plastids were precipitated and then washed twice with Reaction buffer (pH 7.9; 30 mm HEPES‐KOH, 330 mm sorbitol, 2 mm EDTA, 1 mm MgCl_2_, 1 mm MnCl_2_, 10 mm NaHCO_3_ (for acetate labeling), 0.3 mm K_2_HPO_4_).

**Figure 1:**
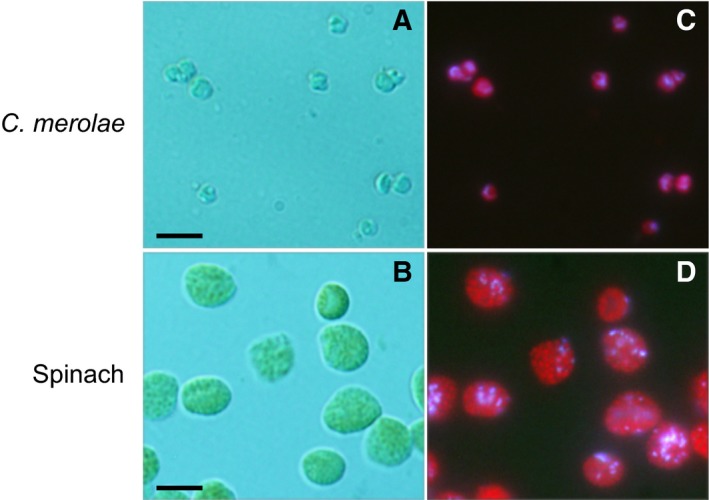
Purity of isolated intact plastids of *Cyanidioschyzon merolae* and intact spinach chloroplasts. (A, B) Nomarski differential interference images. (C, D) Fluorescence images stained with 4′,6‐diamidino‐2‐phenylindole. Bars = 5 μm.

Spinach (*Spinacea oleracea*) was purchased in the local market. Intact spinach chloroplasts were isolated according to the method in Ref. 36 using a Percoll gradient as described above. The purity of isolated *C. merolae* plastids and spinach chloroplasts was checked by fluorescence microscopy (Fig. 1).

### Radioactive isotope labeling

Isolated *C. merolae* plastids (equivalent to 30 mg chlorophyll *a*) were incubated in 600 μL Reaction buffer with sodium [2‐^14^C]acetate (0.3 mm, 10 μCi; 58.8 mCi·mmol^−1^; PerkinElmer, Waltham, MA, USA) or sodium [^14^C] bicarbonate (1 mm, 30 μCi; 58.8 mCi·mmol^−1^; Moravek, Inc., Brea, CA, USA) in 10‐mL glass centrifuge tubes at 38 °C for 5–120 min under light (150 μmol·m^−2^·s^−1^) with vigorous shaking. Labeling experiments were also performed with isolated spinach chloroplasts (equivalent to 250 μg chlorophyll *a* and *b* per reaction) under similar conditions, except that incubation temperature was 25 °C. Reaction was stopped by addition of 3 mL of chloroform/methanol (1 : 2, by volume) for lipid extraction. For analysis of fatty acid export from isolated plastids, the reaction mixture was centrifuged (2000 ***g**,* 30 s) after labeling, and then, total lipids were extracted separately from the supernatant and the precipitate.

The analysis of radiolabeled lipids was performed essentially as described previously 3. Total lipids were extracted according to the method in Ref. 37. Lipid classes were separated by TLC on Silica Gel 60 Plate (Merck, Darmstadt, Germany). The developing solvents were acetone/toluene/methanol/water (8 : 3 : 2 : 1, by volume) for polar lipids and *n*‐hexane/diethyl ether/acetic acid (80 : 30 : 1, by volume) for nonpolar lipids. Fatty acid methyl esters (FAMEs) were prepared by transesterification with 2.5% HCl/methanol (at 85 °C for 2.5 h). After extraction with *n*‐hexane, they were separated by reversed‐phase argentation TLC (RP‐TLC) according to the method in Ref. 38. MGDG that was labeled with [^14^C]bicarbonate was also subjected to transesterification. FAMEs were extracted with *n*‐hexane. Methyl galactoside (plus glycerol) was recovered from the methanol phase and hydrolyzed in 2.4 N hydrochloric acid at 100 °C for 2 h. The reaction mixture was concentrated by evaporation, applied to TLC plates (Silica Gel 60 Plate), and then developed with chloroform/methanol/water (15 : 10 : 2, by volume). Radioactivity on the TLC plates was detected by autoradiography and quantified by liquid scintillation counting.

### Stable isotope labeling

Isolated *C. merolae* plastids (100 mg chlorophyll *a*) were incubated in 1‐mL Reaction buffer with sodium [2‐^13^C] acetate (2 mm; 99% ^13^C; Cambridge Isotope Laboratories, Inc., Tewksbury, MA, USA) or sodium [^13^C] bicarbonate (10 mm; 99% ^13^C; Cambridge Isotope Laboratories, Inc.) at 38 °C for 60 min under light (150 μmol·m^−2^·s^−1^) with vigorous shaking. For comparison, *C. merolae* cells (OD_750_ = 1) were incubated in 1 mL 2 × Allen's medium with sodium [^13^C] bicarbonate (10 mm) under identical conditions. After labeling with [2‐^13^C]acetate, total lipids were extracted and analyzed essentially as described above for radiolabeled lipids. The analysis of ^13^C labeled MGDG was performed as described previously 39. Briefly, labeled MGDG was subjected to methanolysis, and FAMEs were extracted with *n*‐hexane. Methyl galactoside and glycerol were recovered from the methanol phase and trimethylsilylated with BSTFA kit (Tokyo Kasei, Tokyo, Japan). FAMEs and trimethylsilylated sugars were analyzed by GC‐MS according to the procedures in 40. Mass spectral data were processed with the c13dist software 39 (this software is available from http://nsato4.c.u-tokyo.ac.jp/old/C13dist.html).

### Subcellular localization analysis

Subcellular localization of enzymes involved in UDP‐galactose synthesis was analyzed by the GFP‐fusion technique as described in Ref. 5. Briefly, a DNA fragment encoding an *N*‐terminal enzyme sequence was cloned into pCEG1 vector. Table 1 lists primers used in this study. Each GFP construct was transformed into *C. merolae* cells for transient expression. Because GFP fluorescence was faint, transformed cells were fixed and immunostained with anti‐GFP antibody, and then observed with a fluorescence microscope.

**Table 1:** List of primers used in subcellular localization analysis

Locus tag	Primer name	Sequence of primer^a^ (5′ to 3′)	Cloned length (bp)	Full length of enzyme (aa)
CMS159C (UGP1)	CMS159C‐F	TCGTTGACCTCTAGAatgcctttggtgcgaaccag	327	500
	CMS159C‐R	CATGGATCCTCTAGActtcagtacagcgacgcggc		
CMA041C (UGE)	CMA041C‐F	TCGTTGACCTCTAGAatggatcatacgaaaaggat	174	355
	CMA041C‐R	CATGGATCCTCTAGAccgctgctcactggccccgg		
CMO263C (PHD1)	CMO263C‐F	TCGTTGACCTCTAGAatgttcgtagcgcttgcgct	363	370
	CMO263C‐R	CATGGATCCTCTAGAaccgtaagttgcggcatccg		

^a^The uppercase letters within primer sequences are pCEG1 vector sequences required for the recombination‐mediated cloning using the In‐Fusion Cloning Kit (Takara Bio USA, Mountain View, CA, USA).

## Results

### Incorporation of labeled bicarbonate into MGDG in isolated *C. merolae* plastids

**Figure 2:**
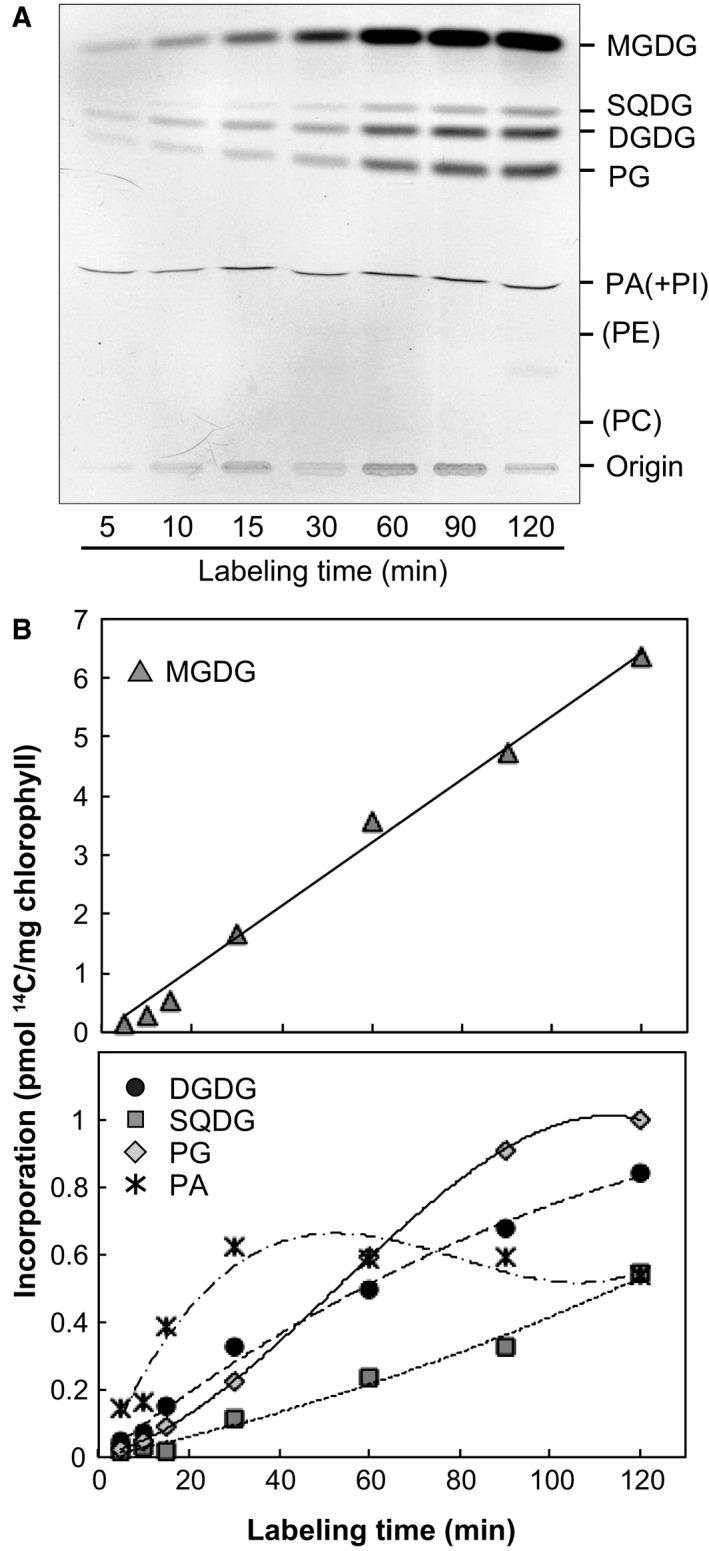
Incorporation of [^14^C]bicarbonate into polar lipids in isolated plastids. (A) Autoradiogram of polar lipids separated by TLC after labeling with [^14^C]bicarbonate. Position of representative lipid classes found in the cell is shown on the right. (B) Time course of incorporation of [^14^C]bicarbonate into polar lipids. Labeling experiments were repeated three times, and representative results are shown. We show here ‘PA(+PI)', because PA and phosphatidylinositol (PI) are not separated by the TLC system (the first dimension in the two‐dimensional system in ref. 7) that we used in the present study, but PI is not expected to be a component of isolated plastids.

Isolated plastids were labeled with [^14^C]bicarbonate under photosynthetic conditions at 38 °C. We used this temperature for incubation, a little lower than the growth temperature (40 °C or 42 °C), because of the limitation of our apparatus for incubation. But this temperature was well within the range of growth temperatures of this organism (from about 25 °C to 46 °C). Total lipids were extracted after labeling for 5–120 min and then fractionated by TLC (Fig. 2A). MGDG was the major lipid class that incorporated radioactivity (Fig. 2B). Radioactivity was also detected in other polar lipids, such as DGDG, SQDG, PG, and phosphatidic acid (PA) (Fig. 2A). Under similar conditions but at 25 °C, [^14^C]bicarbonate was hardly incorporated into polar lipids in spinach chloroplasts as already known (data not shown). Specific radioactivity (proportion of ^14^C in total carbon) of MGDG synthesis was 0.208 × 10^−6^, which was about 2−9 times higher than that in other lipid classes (Table 2). The low rate of synthesis estimated above (compare with the results with ^13^C, see below) was supposed to be a result of very low concentration of radioactive substrates in the incubation medium.

**Table 2:** Incorporation of [^14^C]bicarbonate into plastid membrane lipids in isolated plastids after 1‐h labeling

Lipid	Incorporation of [^14^C]bicarbonate (pmol ^14^C·mg^−1^ chlorophyll·h^−1^)	Lipid content^a^ (nmol lipid·mg^−1^ chlorophyll)	Lipid carbon content (μmol C·mg^−1^ chlorophyll)	Specific radioactivity^b^ (×10^−6^)
	[A]	[B]	[C]	[D]
MGDG	3.58 ± 0.59	401	17.2	0.208
DGDG	0.50 ± 0.23	439	21.5	0.023
SQDG	0.24 ± 0.03	234	10.1	0.023
PG	0.60 ± 0.08	159	6.36	0.094

Calculation: [C] = (carbon number of lipid) × [B]/1000; [D] = [A]/[C]. Carbon numbers: MGDG 43, DGDG, 49, SQDG, 43, PG 40 (assuming C18/C16 species). ^a^Calculated from data in ref. 6. ^b^Specific radioactivity represents proportion of labeled carbon, ^14^C/(^14^C + ^12^C).

**Figure 3:**
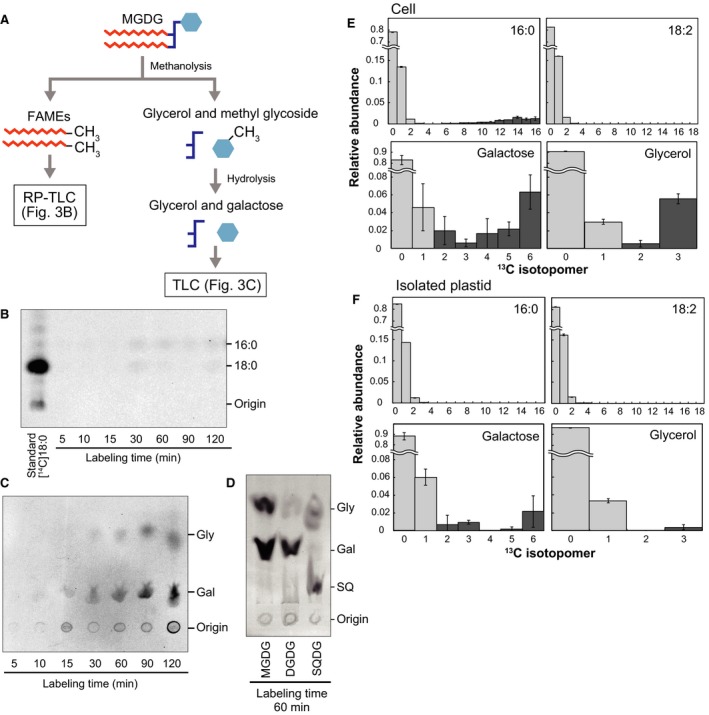
Incorporation [^14^C] and [^13^C] bicarbonate into MGDG in isolated plastids. (A) Flow diagram of analysis of radioactive MGDG. (B) Detection of radioactivity in FAMEs. (C) Detection of radioactivity in galactose and glycerol. (D) Detection of radioactivity in polar groups of DGDG and SQDG. Gal, galactose; Gly, glycerol; SQ, sulfoquinovose. Radiolabeling experiments were repeated three times, and representative results are shown. (E, F) Isotopomer distribution of MGDG synthesized after [^13^C]bicarbonate labeling in intact cells (E) and isolated plastids (F). Unlabeled (or natural abundance) and labeled ^13^C isotopomer populations are shown in light gray and dark gray, respectively. The error bars represented the SD (*n* = 3).

To locate labeled carbons within the MGDG molecule, polar and nonpolar groups were separated by methanolysis, and then, methyl galactoside was hydrolyzed to galactose (Fig. 3A). FAMEs and sugars were separated by RP‐TLC and TLC, respectively. The results indicated that the radioactivity was localized to both glycerol and galactose (Fig. 3C), whereas FAMEs were not labeled significantly (Fig. 3B). This suggests that isolated plastids contain the entire pathway that enables incorporation of photosynthetically fixed carbon into the polar groups of MGDG. In contrast, photosynthate was not efficiently used for the synthesis of fatty acids, as expected from the fact that phosphoglycerate mutase and enolase are not present in isolated plastids. The polar groups of other glycolipids, namely DGDG and SQDG, were also labeled with [^14^C]bicarbonate (Fig. 3D). In DGDG, galactose was labeled with [^14^C]bicarbonate, but glycerol was not labeled significantly, suggesting that radiolabeled DGDG was synthesized from existing, unlabeled MGDG and labeled UDP‐galactose.

We compared labeling of MGDG in isolated plastids and intact cells using a stable isotope ^13^C. Stable isotope was used at a high, physiological concentration to estimate natural rate of synthesis. We determined the distribution of ^13^C isotopomers in individual parts of molecules using the c13dist software 39. At the same time, we characterized the ^13^C isotopomer distribution using an intrinsic variable, isotopic abundance *p*, and distinguished unlabeled (or natural abundance) and labeled isotopomers (for theoretical, and computational details, see Supporting Information in the ref. 39).

In intact cells, highly labeled ^13^C isotopomers were detected in 16:0, galactose and glycerol after labeling for 1 h (Fig. 3E). Galactose also contained less densely labeled population with *p* < 0.5 corresponding to isotopomers 2 and 3, which are likely to be products of condensation of unlabeled and labeled C3 units. To avoid complication, we only used the ^13^C isotopomers with *p* > 0.5 as ‘highly labeled' in the current study. In isolated plastids, galactose and glycerol were labeled with ^13^C, but no labeled ^13^C isotopomers were detected in both 16:0 and 18:2 (Fig. 3F), or other minor fatty acids.

In intact cells, turnover rate of galactose (0.092 h^−1^) was higher than that of glycerol (0.063 h^−1^) and 16:0 (0.073 h^−1^, Table 3). These values were slightly higher than the specific growth rate (0.058 h^−1^: equivalent to the doubling time, 12 h). The excess rate represents degradation rate: namely, [turnover rate] = [specific growth rate] + [degradation rate]. Although exchange of galactose in MGDG has not been reported, the results could indicate rapid exchange of galactose moiety of MGDG.

**Table 3:** Incorporation of [^13^C]bicarbonate into MGDG. nd, not detected

	Content^a^ (nmol·mg^−1^ chlorophyll)	Intact cell			Isolated plastid		
		Relative content of highly labeled isotopomers (%)	Incorporation rate (nmol·mg^−1^ chlorophyll·h^−1^)	Turnover rate (h^−1^)	Relative content of highly labeled isotopomers (%)	Incorporation rate (nmol·mg^−1^ chlorophyll·h^−1^)	Turnover rate (h^−1^)
	[A]	[B]	[C]	[D]	[E]	[F]	[G]
Glycerol	400.92	6.12 ± 0.40	24.55	0.063	0.55 ± 0.22	2.21	0.006
Galactose	400.92	8.80 ± 0.02	35.28	0.092	3.43 ± 0.79	13.63	0.035
16:0	294.44	7.08 ± 2.41	20.85	0.073	nd	nd	nd
18:2	488.41	nd	nd	nd	nd	nd	nd

Turnover rate was estimated from the labeling rate rather than decay rate, assuming a steady‐state situation of a metabolite pool having an input and an output. Highly labeled fraction with *p* > 0.5 was used to estimate incorporation rate and turnover rate. Calculation: [C] = [A] × [B]/100; [F] = [A] × [E]/100; [D] = −ln(1 − [B]/100); [G] = − ln(1 − [E]/100). ^a^Calculated from data in ref. 6.

In isolated plastids, the proportion of highly labeled glycerol (0.55%) was smaller than that of highly labeled galactose (3.43%: total labeled population including isotopomers 2 and 3 was 4.96%, Table 3). Turnover rate of galactose (0.035 h^−1^) was about six times higher than that of glycerol (0.006 h^−1^, Table 3). The labeling of galactose in MGDG can be accounted for by the transfer of newly synthesized galactose to unlabeled pool of DAG remaining in the plastids after isolation. The labeling of glycerol indicates *de novo* synthesis of MGDG, namely, that a small proportion of MGDG was synthesized from newly synthesized galactose and glycerol and unlabeled pool of fatty acids remaining in the plastids. The small size of this pool might limit the rate of *de novo* MGDG synthesis in isolated plastid system. These results suggest that a pathway of providing UDP‐galactose must be present within the plastid in *C. merolae*.

### Absence of *UGP3* homolog encoding plastid‐type UDP‐glucose pyrophosphorylase in red algae

**Figure 4:**
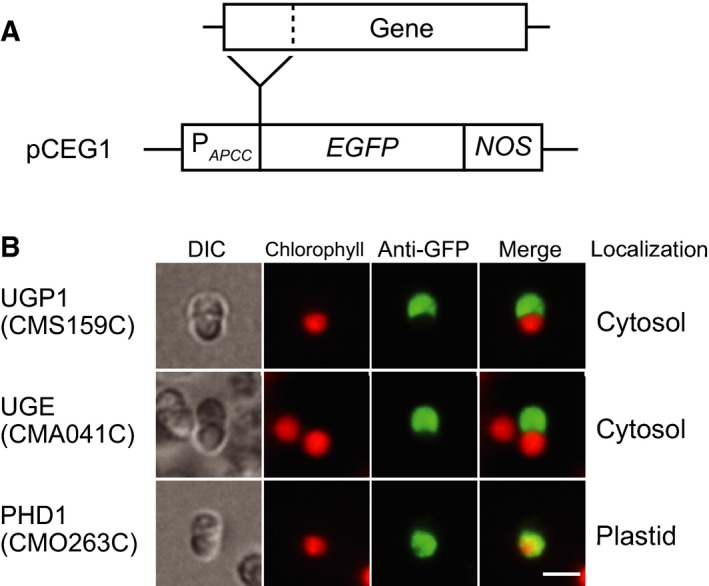
Subcellular localization of enzymes involved in UDP‐galactose synthesis. (A) Schematic diagram of GFP constructs. A DNA fragment of the *N*‐terminal peptide of each enzyme was inserted into pCEG1 vector and cloned. GFP‐fusion proteins were transiently expressed under the control of *APCC* promoter in *Cyanidioschyzon merolae* cells. *EGFP*; enhanced green fluorescence protein gene, *NOS*; nopaline synthase gene terminator, P_*APCC*_; promoter of *APCC* gene of *C. merolae*. (B) Immunofluorescence micrographs of *C. merolae* cells transiently expressing GFP‐fusion protein. Enzyme names are listed in Table 4. Bar = 2 μm.

**Figure 5:**
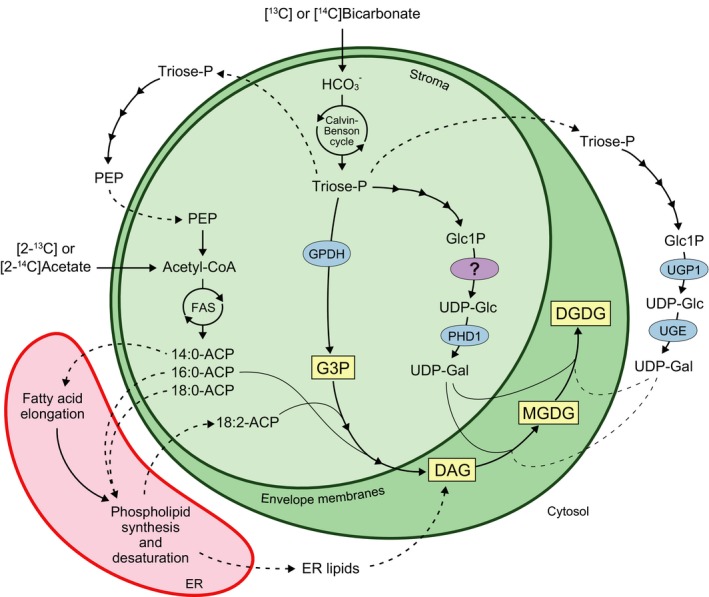
Working metabolic map of galactolipid synthesis in *Cyanidioschyzon merolae*. UDP‐galactose is synthesized by two pathways in plastid and cytosol. Dashed lines indicate transport of substrates between plastid and other compartments. UDP‐galactose synthesized by the two pathways is used as a substrate for galactolipid synthesis. G3P is synthesized from triose‐phosphate by plastid‐type glycerol 3‐phosphate dehydrogenase (GPDH; CMR476C, 33). Two cytosolic enzymes, phosphoglycerate mutase and enolase, are required to synthesize acetyl‐CoA from triose‐phosphate (as in land plants). Glc1P, glucose‐1‐phosphate; PEP, phosphoenolpyruvate; Triose‐P, triose‐phosphate; UDP‐Gal, UDP‐galactose; UDP‐Glc, UDP‐glucose. Enzyme names involved in UDP‐galactose synthesis are shown in Table 4.

Comparative genomic analysis using the Gclust database (dataset Gclust2016R, 41) identified enzymes involved in UDP‐galactose synthesis, such as UDP‐glucose pyrophosphorylase and UDP‐glucose epimerase in *C. merolae*. *C. merolae* has homologs of *UGP1* encoding cytosolic type of UDP‐glucose pyrophosphorylase (CMS159C) and *UGE* encoding UDP‐glucose epimerase (CMA041C) (Table 4). *PHD1* homolog (CMO263C) encoding plastid‐type UDP‐glucose epimerase was detected (Table 4). But no homolog of *UGP3* encoding plastid‐type UDP‐glucose pyrophosphorylase was detected in *C. merolae* and other red algal genomic data. Additionally, cyanobacterial UDP‐glucose pyrophosphorylase, such as GalU and CugP 42, was not detected in red algae. Next, we analyzed subcellular localization of these enzymes using GFP‐fusion technique (Fig. 4A). GFP‐fusion proteins of two homologs of cytosolic type enzymes, UGP1 (CMS159C) and UGE (CMA041C) were localized to the cytosol (Fig. 4B). GFP‐fusion protein of PHD1 homolog (CMO263C) was targeted to the plastid (Fig. 4B). As shown by a question mark in the current working metabolic map of galactolipid synthesis in *C. merolae* (Fig. 5), an enzyme that catalyzes the synthesis of UDP‐glucose must be present in the red algal plastids to account for the labeling results (see Discussion section). In other words, we have to suppose that *C. merolae* has two pathways of UDP‐galactose synthesis, one in the cytosol and the other in the plastid (Fig. 5).

**Table 4:** Summary of enzymes involved in UDP‐galactose synthesis in land plants, green algae, and red algae. nd, not detected

Enzyme	EC number	Gene name	Land plants	Green algae	Red algae				
			*Arabidopsis thaliana*	*Chlamydomonas reinhardtii*	*Cyanidioschyzon merolae*	*Porphyridium purpureum*	*Galdieria sulphuraria*	*Chondrus crispus*	*Pyropia yezoensis*
UDP‐Glucose pyrophosphorylase	2.7.7.9	*UGP1/2*	AT3G03250 (UGP1) AT5G17310 (UGP2)	Cre04.g229700.t1.2	CMS159C (UGP1)	3396.11 3601.4	EME32720	CDF37168	c25732_g6344 c29138_g7162
		*UGP3*	AT3G56040 (UGP3)	Cre12.g554250.t1.1	nd	nd	nd	nd	nd
UDP‐Glucose epimerase	5.1.3.2	*UGE1‐5*	AT1G12780 (UGE1) AT4G23920 (UGE2) AT1G63180 (UGE3) AT1G64440 (UGE4) AT4G10960 (UGE5)	g4686.t1	CMA041C (UGE)	2302.7	EME31528	CDF32840	c20015_g4925
		*PHD1*	AT2G39080 (PHD1)	Cre13.g608000.t1.2	CMO263C (PHD1)	3440.13	EME28001	CDF37085	c4063_g891 c6457_g1467

### Synthesis of saturated fatty acids in isolated plastids of *C. merolae*

**Figure 6:**
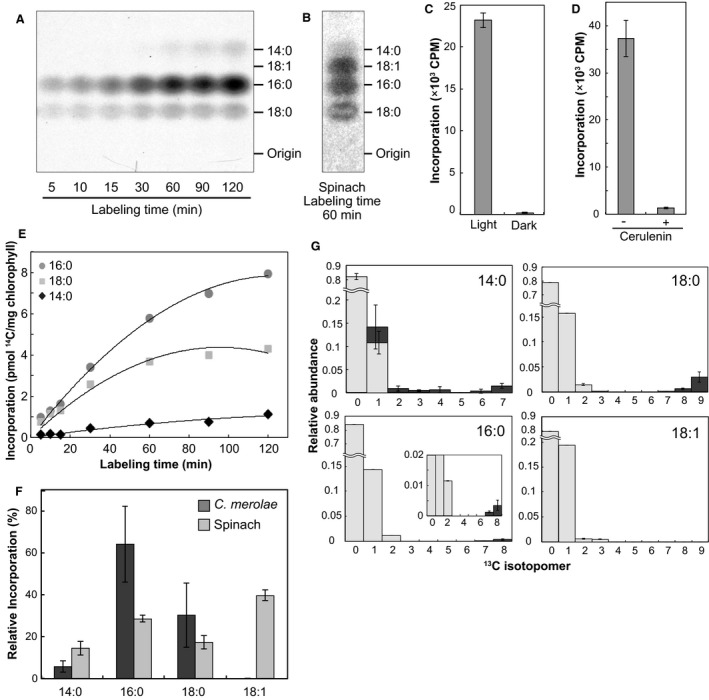
Incorporation of [2‐^14^C] and [2‐^13^C]acetate into fatty acids in isolated *Cyanidioschyzon merolae* plastids and spinach chloroplasts. (A, B) RP‐TLC separation of FAMEs derived from the total lipids after incorporation of [2‐^14^C]acetate in isolated *C. merolae* plastids (A) and spinach chloroplasts (B). (C, D) Effect of dark (C) and cerulenin addition (D) on the incorporation of [2‐^14^C]acetate into fatty acids in isolated plastids of *C. merolae*. (E) Time course of incorporation of [2‐^14^C]acetate into FAMEs in *C. merolae* plastids. (F) Comparison of fatty acid labeling, after 60‐min incorporation of [2‐^14^C]acetate in isolated *C. merolae* plastids and spinach chloroplasts. Radiolabeling experiments were repeated three times, and representative results are shown. (G) Incorporation of [2‐^13^C]acetate (2 mm) into fatty acids of total lipids in isolated plastids for 1 h. Unlabeled (or natural abundance) and labeled populations of ^13^C isotopomers are shown in light gray and dark gray, respectively. The inset shows an enlargement of ^13^C isotopomer distribution. The error bars represented the SD (*n* = 3).

Labeled acetate was used to study fatty acid synthesis in isolated plastids. Intact *C. merolae* plastids were incubated with [2‐^14^C]acetate for 5−120 min in the light at 38 °C. Total lipids were extracted and subjected to methanolysis, and then, FAMEs were separated by RP‐TLC. Radioactive spots of myristic acid (14:0), 16:0, and 18:0 were detected by autoradiography, but no spot assignable to unsaturated fatty acids, such as 18:1, was detected (Fig. 6A). This incorporation was dependent on light during the incubation (Fig. 6C) and was efficiently inhibited by cerulenin, an inhibitor of 3‐ketoacyl‐(acyl‐carrier‐protein) synthase I/II (or KAS I/II, Fig. 6D).

In spinach chloroplasts, on the other hand, 16:0, 18:0, and 18:1 were labeled as a result of incorporation of [2‐^14^C]acetate (Fig. 6B). These spots were scraped off the plates, and radioactivity was quantified by liquid scintillation counting (Fig. 6E,F). 16:0 was a major labeled fatty acid in isolated *C. merolae* plastids, equivalent to about 8 pmol [2‐^14^C]acetate per mg chlorophyll *a* after labeling for 120 min (Fig. 6E). In contrast, 18:1 was the major labeled fatty acid in spinach chloroplasts after labeling for 60 min (Fig. 6F). Incorporation rate of [2‐^14^C]acetate into 16:0 was 5.77 pmol·mg^−1^ chlorophyll h^−1^ in *C. merolae* plastids, which was higher than that of other fatty acids (Table 5). Nevertheless, specific radioactivity was higher in 14:0 and 18:0, reflecting different turnover rates.

**Table 5:** Incorporation of [2‐^14^C]acetate into fatty acids by isolated plastids for 1 h labeling. FA, fatty acid

Fatty acid	Incorporation of [^14^C]acetate (pmol ^14^C·mg^−1^ chlorophyll·h^−1^)	Fatty acid content^a^ (nmol FA·mg^−1^ chlorophyll)	Fatty acid content in acetate units (nmol C2·mg^−1^ chlorophyll)	Specific radioactivity^b^ (×10^−6^)
	[A]	[B]	[C]	[D]
14:0	0.70 ± 0.50	6.19	43.3	16.2
16:0	5.77 ± 1.40	1078	8624	0.669
18:0	3.68 ± 3.00	293	2637	1.396

Calculation: [C] = [B] × (C2 units); [D] = 1000 × [A]/[C]. C2 units: 7 for 14:0, 8 for 16:0, 9 for 18:0. ^a^Calculated from data in ref. 6. ^b^Specific radioactivity represents proportion of labeled carbon, ^14^C/(^14^C + ^12^C).

To confirm the synthesized fatty acids in isolated plastids, we carried out labeling experiments using [2‐^13^C]acetate. If fatty acids are synthesized *de novo* from [2‐^13^C]acetate by FAS, up to a half of ^12^C in the fatty acids (even‐numbered carbons) should be replaced by ^13^C. The result showed that highly labeled ^13^C isotopomers (containing 6, 7, 8, and 9 isotopes) were detected in saturated fatty acids, such as 14:0, 16:0, and 18:0 (Fig. 6G). The isotopomer 1 in 14:0 could represent elongation product incorporating only one C2 unit, but this should be interpreted carefully because the error range was large. The turnover rates of 14:0 (0.023 h^−1^) and 18:0 (0.038 h^−1^) were higher than that of 16:0 (0.005 h^−1^) and fairly lower than the specific growth rate (0.058 h^−1^) as described above (Table 6). The very low turnover rate of 16:0 could result from the *in vitro* situation, in which no further metabolism of 16:0 (mainly lipid biosynthesis) is arrested in isolated plastids. Apparently higher content of labeled population in 18:0 reflects its smaller pool size, allowing a faster turnover in restricted, *in vitro* situation. No newly synthesized ^13^C isotopomers were detected in 18:1 (Fig. 6G) and 18:2 (data not shown). This was consistent with the results of radiolabeling. These results suggest that *C. merolae* plastid synthesizes solely saturated fatty acids, in contrast with the plastids in land plants.

**Table 6:** Incorporation of [2‐^13^C]acetate into fatty acids. nd, not detected

Fatty acids	Fatty acid content^a^ (nmol·mg^−1^ chlorophyll)	Relative content of labeled isotopomers (%)	Incorporation rate (nmol·mg^−1^ chlorophyll·h^−1^)	Turnover rate (h^−1^)
	[A]	[B]	[C]	[D]
14:0	6.19	2.28 ± 0.82	0.14	0.023
16:0	1078.3	0.49 ± 0.24	5.25	0.005
18:0	293.2	3.72 ± 0.45	10.91	0.038
18:1	360.9	nd	nd	nd

Highly labeled fraction with *p* > 0.5 was used to estimate incorporation rate and turnover rate. Calculation: [C] = [A] × [B]/100; [D] = −ln(1 − [B]/100). ^a^Calculated from data in ref. 6.

### Export of saturated fatty acids

**Figure 7:**
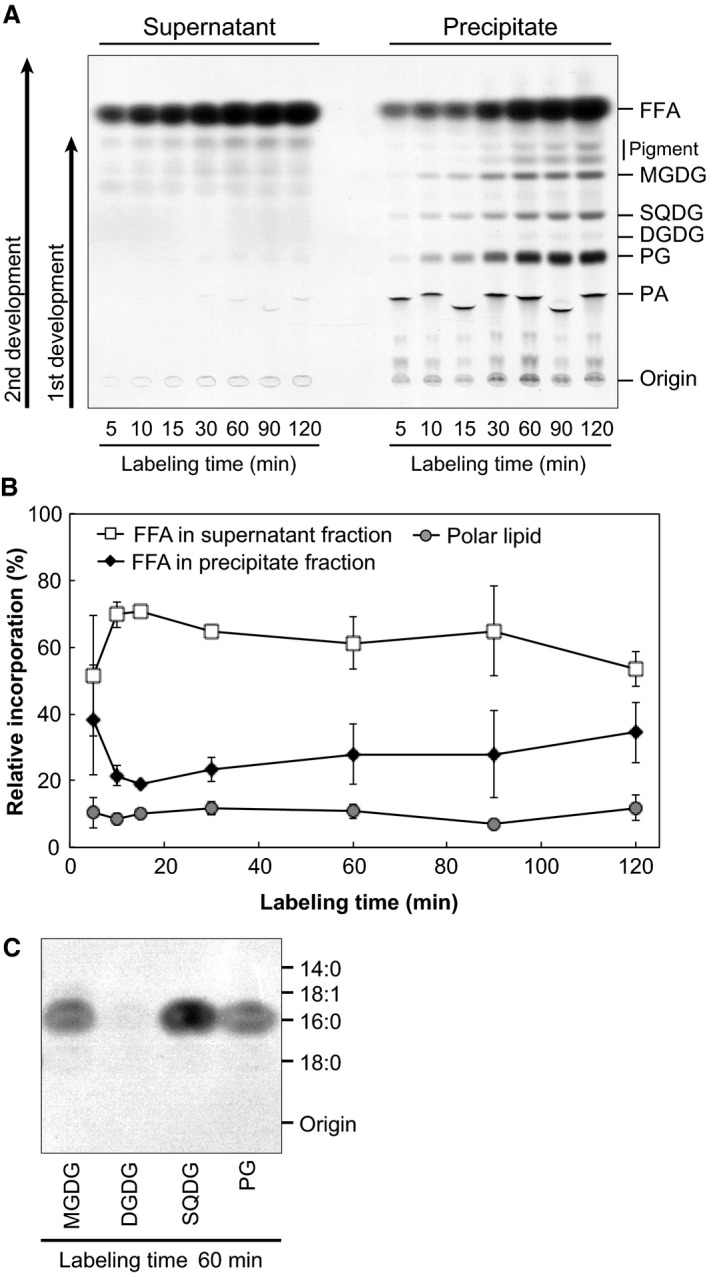
Fatty acid export in isolated plastids from *Cyanidioschyzon merolae*. (A) Two‐step TLC separation of lipid classes in the supernatant and precipitate fractions. First, polar lipids were separated by developing halfway the TLC plate with acetone/toluene/methanol/water (8 : 3 : 2 : 1, by volume). After drying, nonpolar lipids were further developed to the top with *n*‐hexane/diethyl ether/acetic acid (80 : 30 : 1, by volume). (B) Time course of relative incorporation of [2‐^14^C]acetate into polar lipids and FFAs in supernatant and precipitate fractions. The error bars represented the SD (*n* = 3). (C) Incorporation of [2‐^14^C]acetate into FAMEs of plastid membrane lipids. After methanolysis, FAMEs were extracted and fractionated by RP‐TLC. Labeling experiments were repeated three times, and representative results are shown.

Next, we analyzed the molecular form of labeled fatty acids by TLC. Total lipids were extracted from the supernatant and the precipitate of the reaction mixture after labeling with [2‐^14^C]acetate, and then, lipid classes were fractionated by TLC (Fig. 7A). The spots of free fatty acids (FFAs) were detected from both supernatant and precipitate fractions. In polar lipids, radioactivity was also detected in MGDG, SQDG, PG, and PA from the precipitate fraction. Figure 7B compares incorporations of [2‐^14^C]acetate into supernatant FFAs, precipitate FFAs, as well as precipitate polar lipids. About 70% of newly synthesized fatty acids were detected in supernatant as FFAs, whereas relative incorporation in polar lipids was about 10%. Additionally, 16:0 was the sole labeled fatty acids incorporated into the polar lipids after labeling with [2‐^14^C]acetate (Fig. 7C). These results suggest that most newly synthesized fatty acids including all 14:0 and 18:0 (about 10% and 30% in total) and 50% of 16:0 were exported out of plastids.

## Discussion

In the present study, we performed labeling experiments using isolated plastids of *C. merolae* to obtain insights into the synthesis of fatty acids and lipids in red algal plastids. We used both radioisotope and stable isotope to characterize labeled molecules qualitatively and quantitatively. Stable isotope labeling clearly identified the molecules synthesized *de novo* having a high isotopic abundance value, *p*.

### Supply of polar groups in plastid lipids

Radiolabeling experiments using [^14^C]bicarbonate showed that the major labeled lipid was MGDG in isolated *C. merolae* plastids (Fig. 2), which was not the case in spinach chloroplasts. [^14^C]bicarbonate was mainly incorporated into the polar groups of MGDG (Fig. 3A–C). This was supported by comparing labeling patterns of MGDG in isolated plastids and intact cells using a stable isotope, [^13^C]bicarbonate (Fig. 3E,F). These results suggest that UDP‐galactose and glycerol 3‐phosphate (G3P) are synthesized from photosynthates within the plastid of *C. merolae* (Fig. 5). We performed comparative genomic analysis and localization analysis of putative enzymes involved in UDP‐galactose synthesis in *C. merolae* (Table 4, Fig. 4). The presence of cytosolic type UDP‐glucose pyrophosphorylase (UGP1) and UDP‐glucose epimerase (UGE), and plastid‐type UDP‐glucose epimerase (PHD1) suggests that *C. merolae* has two pathways of UDP‐galactose synthesis, one in the plastid and the other in the cytosol (Fig. 5). In a previous study, both MGDG and DGDG were labeled when isolated plastids of *C. merolae* were incubated with UDP‐[^14^C]galactose 3. This suggests that UDP‐galactose could also be supplied from the cytosol to synthesize galactolipids in *C. merolae*. (Fig. 5). The cyanobacterial DGDG synthase in *C. merolae*, DgdA, is also likely to accept UDP‐galactose from the cytosol and within the plastid, because labeling of the galactose moiety of DGDG was detected in isolated plastids, although the absolute rate of labeling was low (Fig. 3D).

A question remains: Namely, no homolog of plastid‐type UDP‐glucose pyrophosphorylase was detected in red algae by comparative genomic analysis (Table 4). Not only in *C. merolae*, but also in all red algae analyzed, no homolog of this enzyme was detected. We consider that an alternative UDP‐glucose pyrophosphorylase might be present in red algal plastids for the following reasons: (a) The galactose moiety of MGDG was labeled with [^13^C] or [^14^C]bicarbonate in isolated plastids lacking cytosolic compartment. (b) Polar groups of SQDG were also labeled with [^14^C]bicarbonate in isolated plastid (Fig. 3D), suggesting that UDP‐sulfoquinovose is synthesized from radiolabeled UDP‐glucose within the isolated plastids.

### Synthesis of acyl groups in red algal plastids

The acyl groups of MGDG were not labeled significantly with [^14^C] or [^13^C]bicarbonate in isolated *C. merolae* plastids (Fig. 3B,F). Since *C. merolae* does not have phosphoglycerate mutase and enolase in the plastid, as in land plants 31, we consider that acetyl‐CoA required for fatty acid synthesis was not synthesized from [^14^C]bicarbonate within isolated plastids devoid of cytosol fraction (Fig. 5).

In the labeling experiments with [2‐^14^C]acetate, isolated plastids of *C. merolae* synthesized saturated fatty acids, 16:0 and 18:0, but not 18:1 (Fig. 6A,E,F), which is the major fatty acid synthesized in isolated spinach chloroplasts. This was supported by labeling experiments using [2‐^13^C]acetate (Fig. 6G). These results suggest that the desaturation of 18:0 does not take place in the plastid of *C. merolae*, as expected from the lack of stearoyl‐ACP desaturase. A previous study reported that a single KAS might be involved in the fatty acid synthesis in *C. merolae* plastid 5. In land plants, fatty acid synthesis involves three isoforms of KAS (KAS I/II/III) having different substrate specificities 43, 44. In *C. merolae*, 16:0 and 18:0 were major fatty acids synthesized in isolated plastids (Fig. 6E,F). Additionally, fatty acid synthesis activity in isolated *C. merolae* plastids was efficiently inhibited by cerulenin, which is a KAS I/II inhibitor (Fig. 6D). These results suggest that a single KAS in *C. merolae* is likely to catalyze all condensing reactions involved in fatty acid with a broad substrate specificity compared with plant KAS (I, II, and III).

The majority of products from fatty acid synthesis in isolated plastids were detected as FFAs (Fig. 7A). About 70% of labeled fatty acids were found in the supernatant fraction of the reaction mixture (Fig. 7B), suggesting that isolated *C. merolae* plastids actively export newly synthesized fatty acids. On the other hand, about 10% of labeled fatty acids were found in polar lipids, such as MGDG, SQDG, and PG (Fig. 7A,B). Practically no labeled DGDG was detected after labeling with [2‐^14^C]acetate for 2 h (Fig. 7A). Slow labeling with [2‐^14^C]acetate of this lipid class was also observed in the labeling experiments using intact cells of *C. merolae* 3. [^14^C]Bicarbonate, however, was incorporated into the galactose moiety of DGDG in isolated *C. merolae* plastids (Fig. 3D), suggesting that the galactosylation from MGDG to DGDG proceeds in isolated plastids. In *C. merolae*, the supply of 18:2 from ER is important for the coupled pathway 3. It is likely that plastid membrane lipid synthesis is not active in isolated plastids due to the lack of 18:2 supply from ER, which resulted in higher accumulation of FFAs outside the plastids. The low turnover rate obtained with ^13^C corroborates this idea. Radiolabeled MGDG, SQDG, and PG incorporated solely 16:0 (Fig. 7C), suggesting that the acyltransferase in the coupled pathway might have a rather stringent substrate specificity. The high specific radioactivity (16.2 × 10^−6^, Table 5) and the high turnover rate of 14:0 (0.023 h^−1^, Table 6) suggest that 14:0 is turned over rapidly, keeping a small pool of 14:0 in *C. merolae* (about 0.1–1%, 6). It seems that newly synthesized 14:0 might be elongated to 16:0 by fatty acid elongation system in ER (Fig. 5).

### General model of red algal lipid biosynthesis

**Figure 8:**
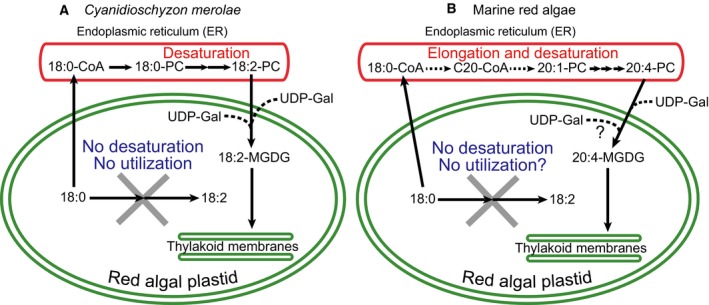
Essential similarity of lipid biosynthesis in *Cyanidioschyzon merolae* (A) and marine red algae (B). In both models, ‘indigenous C18 fatty acids are neither desaturated nor directly utilized within the plastid' is a common principle. Panel A summarizes major findings in the present study. For simplicity, only C18 fatty acids are shown. The 18:0 synthesized by the fatty acid synthase in the plastid is exported to the cytosol and subsequently incorporated into phosphatidylcholine (PC) via CoA thioester in the ER. Then, the acyl groups are desaturated to produce 18:2. Genomic data indicate that desaturation of 18:0‐CoA to 18:1‐CoA is also possible in ER. In this case, 18:1‐PC is formed, and then desaturated. Panel B is a model in marine red algae, such as *Pyropia* and *Porphyridium* mainly based on 1, which contain 20:4 or 20:5 as major fatty acids in MGDG and other plastid lipids. For simplicity, only 20:4 and MGDG are shown. The 18:0 synthesized within the plastid is exported and ultimately incorporated into PC. During the process, desaturation and elongation take place in acyl‐CoA, but the exact sequence of process is not clear. 20:1 is further desaturated to 20:4, and possibly 20:5 in either PC or CoA. Finally, 20:4 (or 20:5) is incorporated into plastid lipids. The last step of desaturation is also likely to occur in the plastid. Apparently very different composition of fatty acids in MGDG or other plastid lipids can be understood according to the common principle ‘no desaturation or direct utilization of C18 acids synthesized in the plastid'. UDP‐Gal, UDP‐galactose.

The findings of the present study can be understood in the general framework of red algal lipid biosynthesis as announced in Introductory text. *C. merolae* might have two pathways of UDP‐galactose synthesis in both cytosol and plastid, which is not the case in land plants. Saturated fatty acids are the only products of fatty acid synthesis in plastids and rapidly exported from plastids. Comparative genomics suggested that these findings are expected to be true in all red algae. We know that *C. merolae* does not synthesize polyunsaturated fatty acids, whereas marine macrophytic red algae are rich in 20:4 and 20:5. This apparent discrepancy can be understood by the same principle, namely, that the C18 fatty acids produced in the plastid are neither desaturated nor directly utilized within the plastid. The whole pool of 18:0 is likely to be exported from the plastid. In *C. merolae*, desaturation of 18:0 (exported from the plastid) in the ER is the only way of producing 18:2 which is subsequently imported into the plastid (Fig. 8A). In *Porphyridium* or *Pyropia* 1, elongation and desaturation of 18:0, also expected to be exported from the plastids, produce 20:4 and 20:5, which are subsequently incorporated into the plastids for the synthesis of MGDG, highly enriched in these polyunsaturated fatty acids (Fig. 8B). C18 unsaturated fatty acids are very scarce in these marine red algae. Therefore, ‘indigenous C18 fatty acids are neither desaturated nor directly utilized within the plastid' is the common principle in both *C. merolae* and marine red algae, apparently very different in fatty acid composition. Classical metabolic studies illustrated the pathway of synthesis of 20:4 by starting from 18:1 17, 27, but the starting point could be 18:0 (or 16:0). Curiously, diatoms possess stearoyl‐ACP desaturase 45 in the plastids, which is supposed to result from the secondary endosymbiosis of red algal plastid. The pathway of fatty acid synthesis might be further complicated during the evolution in these algae.

## Conflict of interest

The authors declare no conflict of interest.

## Author contributions

All authors conceived and supervised the study and designed experiments; NM performed experiments; NM and NS wrote the manuscript.
